# Using best-worst scaling to inform policy decisions in Africa: a literature review

**DOI:** 10.1186/s12889-024-20068-w

**Published:** 2024-09-27

**Authors:** Laura K. Beres, Nicola B. Campoamor, Rachael Hawthorn, Melissa L. Mugambi, Musunge Mulabe, Natlie Vhlakis, Michael Kabongo, Anne Schuster, John F. P. Bridges

**Affiliations:** 1 Department of International Health, Johns Hopkins Bloomberg School of Public Health, 615 N Wolfe Street, Office, Baltimore, MD 5032, 21205 USA; 2 Department of Biomedical Informatics, The Ohio State University College of Medicine, 220 Lincoln Tower, 1800 Cannon Drive, Columbus, OH 43210 USA; 3 Center for the Advancement of Team Science, Analytics, and Systems Thinking in Health Services and Implementation Science Research (CATALYST), The Ohio State University, 700 Ackerman Road, Columbus, OH 43202 USA; 4 Department of Global Health, University of Washington, UW Box #351620, Seattle, WA 98195 USA; 5 Centre for Infectious Disease Research in Zambia, Stand 378A / 15, Main Street, P.O. Box 34681, Ibex, Lusaka Zambia

**Keywords:** Best-worst scaling, Measuring priorities, Africa, Stakeholder engagement

## Abstract

**Background:**

Stakeholder engagement in policy decision-making is critical to inform required trade-offs, especially in low-and-middle income settings, such as many African countries. Discrete-choice experiments are now commonly used to engage stakeholders in policy decisions, but other methods such as best-worst scaling (BWS), a theory-driven prioritization technique, could be equally important. We sought to document and explore applications of BWS to assess stakeholder priorities in the African context to bring attention to BWS as a method and to assess how and why it is being used to inform policy.

**Methods:**

We conducted a literature review of published applications of BWS for prioritization in Africa.

**Results:**

Our study identified 35 studies, with the majority published in the past four years. BWS has most commonly been used in agriculture (43%) and health (34%), although its broad applicability is demonstrated through use in fields influencing social and economic determinants of health, including business, environment, and transportation. Published studies from eastern, western, southern, and northern Africa include a broad range of sample sizes, design choices, and analytical approaches. Most studies are of high quality and high policy relevance. Several studies cited benefits of using BWS, with many of those citing potential limitations rather than observed limitations in their study.

**Conclusions:**

Growing use of the method across the African continent demonstrates its feasibility and utility, recommending it for consideration among researchers, program implementers, policy makers, and funders when conducting preference research to influence policy and improve health systems.

**Registration:**

The review was registered on PROSPERO (CRD42020209745).

## Introduction

Health policies govern both health systems hardware (e.g., human resources, finance, medicines and technologies) and software (e.g., values, norms, power dynamics) by constraining or facilitating individual, organizational, and community actions and experiences [1–3]. Additionally, healthcare workers take numerous discretionary decisions each day to translate policy into practice and to fill gaps between policy guidance and implementation realities [4–6]. When guided by evidence, health policies and clinical decision-making facilitate optimized health practices and outcomes [6–8]. However, policy and practice decisions are often made in an evidence void due to a lack of data or poor evidence access and translation, resulting in inefficient, ineffective, or harmful health system outcomes [9–11].

Evidence about the preferences of those affected by health decision-making is particularly limited, but greatly needed for policy and practice. Internationally recognized processes for developing health guidelines and recommendations include incorporating the values and preferences of affected parties, such as patients and healthcare workers, into decision-making [12, 13]. Limited availability of preference-based evidence downgrades the strength of recommendations [13, 14]. Additionally, the welcomed and growing call for person-centered healthcare explicitly requires integration of patient preferences and perspectives into health practice [15, 16]. Across all settings, policy makers must trade off services implemented with available resources. Required trade-offs are often more common and more challenging in more resource-limited settings, such as low-and-middle-income countries. Expanded use of tools to understand and systematically incorporate evidence on the preferences of patients, healthcare workers, and other stakeholders into policies and practices is needed to improve health systems at every level [17–20].

Researchers and practitioners use multiple methods to understand preferences and priorities, including deliberative processes such as testimony or community meetings, qualitative processes such as focus group discussions and interviews, and mixed methods approaches such as human-centered participatory design processes, surveys, Likert scales, and community scorecards. Developing an even broader methodological toolkit allows for more influential data to facilitate policy and practice changes, as teams will be equipped to optimize the methods selected for the target audience and research question. Stated preference methods offer a theory-driven, structured approach to understanding preferences and priorities. They produce interpretable outcomes with clear relevance to the questions of interest. They have been used across various industries, including healthcare, transportation services, and grocery retailing demonstrating their versatility and potential for suitability. However, while a range of preference elicitation methods exist [21–23], discrete choice experiments (DCEs) predominate in published health literature [24]. Recent studies internationally have shown the potential utility and appropriateness of other, lesser known but valuable quantitative stated preference methods, such as best-worst scaling (BWS) [25]. Studies from eastern, western, southern, and northern Africa have demonstrated interesting advances [26–28], but have received less attention than BWS in other regions.

The goal of this study was to document and explore applications of BWS to assess stakeholder priorities in eastern, western, southern, and northern Africa to inform future preference assessment implementation. Such documentation of current BWS in the African setting is an important step in bringing attention to this increasingly important method and to stress that there are other theory-driven alternatives to DCEs – that are now commonly applied in Africa [24]. The review presents study design, methods, quality, and policy relevance from extant studies to enable preference researchers to consider the appropriateness of similar BWS applications in their work. While several international reviews have been conducted on BWS in health [25, 29] and more generally [30], it is important to document how this method is being used in the African context, and what specific role it might have in informing policy there. Furthermore, there has been increased use of these methods in Africa since these previous reviews. It is important that contributions of African preference researchers are well-document to ensure their inclusion in international efforts around preference methods and the presented strengths and weaknesses of these methods are well understood [31].

## Background

Best-worst scaling (BWS) is a choice experiment that is aimed to assess how individiuals or groups prioritize concepts. It offers a theory-driven alternative to descriptive rating, ranking or Likert scale preference measurement, leveraging relative participant ease of selecting extremes compared to mid-range rankings. Several types of BWS exist; however, they all share the same underlying concept. BWS relies on the concept of individuals choosing the ‘best’ and ‘worst’ (or ‘most’ and ‘least’ important) items from a given sub-set of three or more items. Sub-sets of the items are shown repeatedly in different combinations requiring choices of ‘best’ and ‘worst’ within each sub-set. This results in a prioritization of the items. Even if all options are preferred, participants are forced to prioritize among the choices. The purpose is to determine the most and least preferred options of items existing on a subjective, latent value continuum. The application of BWS for prioritizing objects has also been referred to as MaxDiff, object scaling, BWS case 1, or BWS object case. Best-worst responses can also be used in other choice formats that are more similar to DCEs, which are not the focus of this paper [32].

BWS draws on random utility theory to identify perceived importance and priorities among a set of items (known as attributes) of a scenario based on repeat choices. It can estimate the likelihood of preference selection and heterogeneity of preferences between groups. BWS can be used with a relatively small sample size and analyzed with a range of methods including more simple count analyses or more complex probablistic models. The range of analysis approaches makes them particularly useful when working across a broad range of stakeholders, including policy makers, who would want and need to understand how conclusions were drawn. Compared to DCEs, where participants select which of two or more presented profiles (specific item combinations) are preferred, BWS offers more information per choice task (i.e., best and worst choices instead of only best), allowing for a more efficient design with either a smaller sample size or more information per task. BWS may have a lower cognitive burden for participants than DCEs [33, 34]. We refer the reader to additional resources for more detail on the theory, methods, and application of BWS [35, 36].

## Methods

Our review of BWS for prioritization in published research from southern, eastern, western, and northern Africa drew from a broader database of BWS studies identified in previous reviews. While an earlier literature review on all types of BWS choice formats had previously been published [29], our team completed the first systematic review of BWS applications in health published prior to 2022 [25]. We then extended our review (PROSPERO: CRD42020209745) to include publications from any field (e.g., health, business, agriculture, etc.) published prior to 2023 [30]. We have continued to improve this database of articles, utilizing additional search strategies and including previously unidentified relevant articles directly sent to our team. Our database currently has 623 published studies from which we systematically extract data application, development, design, administration/analysis, quality, and policy relevance. The study reported in this paper leverages the most expanded database. Review methods were detailed in prior publications [25].

We included all studies from the database that focused exclusively on, or incorporated into a multi-country study, participants from eastern, western, southern, or northern Africa. We then accessed extracted data in the database for each study to characterize BWS application types, study methods, context, quality, and policy relevance. Specific fields included: study year, country, topic, objective, sample size, perspective (i.e., whose preferences are measured), terminology used to describe BWS type (e.g., object case, MaxDiff), mode of survey administration (e.g., in-person), time frame of prioritization scenario (i.e., past, present, or future), methods of instrument development (e.g., formative research, literature review to determine attributes and survey tool), time frame of prioritization scenario (i.e., past, present, or future), measurement scale, experimental design type, BWS anchor description (i.e., most/least, best/worst), directionality, total number of objects, number of objects per task, number of tasks in the experiment, number of tasks per respondent, analysis approach, and theoretical assumptions [25]. We re-classified database ‘topic’ for three studies from ‘agriculture’ to ‘business’ after reviewing study journal and conclusions. To understand study quality, we utilized the PREFS checklist quality assessment which measures quality and validity of preference studies on a 0–5 point scale, assigning one point for each of the following: **p**urpose of the study clearly defined; **r**espondents similar to non-respondents (sampling); **e**xplanation of preference assessment methods clear; **f**indings reported for all respondents; and significance testing done [37]. We also present the validated subjective quality (range: 1–10) and policy relevance (range: 1–10) scores adjudicated by the prior review.

Identified strengths and weaknesses of BWS were extracted from the studies. This information primarily came from the background, methods, and discussion portions of the studies, specifically when justifying the use of BWS and highlighting any study limitations. Seven domains of strengths and weaknesses were dervied and modified from exisiting best-practice documents for preference research [38, 39].

To highlight the application of BWS and improve understanding of the method, we include a narrative case study description of two of the included manuscripts. We chose health-related studies selected for their high quality (≥ 4 PREFS score) and policy relevance (≥ 7) with illustrative diversity across other factors including country, perspective, design, instrument development, and analysis approach. While diversity in other points such as assumptions, directionality, and heterogeneity analysis are interesting, (See Tables 1, 2 and 3) the selected articles offered rich contrast among the articles in this review.

**Table 1:** Study characteristics of BWS applications in Africa

All Studies	*N* = 35	
Year Published, *n*(%)		
2011–2014	4	(11%)
2015–2018	6	(17%)
2019–2022	18	(52%)
2023	7	(20%)
BWS Description Terminology, *n*(%)		
Best-Worst Scaling	30	(85%)
BWS - Case 1	2	(6%)
MaxDiff	2	(6%)
BWS - Object Case	1	(3%)
Country, *n*(%)		
South Africa	12	(34%)
Kenya	4	(11%)
Niger	4	(11%)
Nigeria	3	(9%)
Ghana	2	(6%)
Sierra Leone	2	(6%)
Benin	1	(3%)
Botswana	1	(3%)
Madagascar	1	(3%)
Mauritania	1	(3%)
Rwanda	1	(3%)
Senegal	1	(3%)
Uganda	1	(3%)
Multiple from West Africa, unspecified	1	(3%)
Literature Topic, *n*(%)		
Agriculture	15	(43%)
Health	12	(34%)
Business	3	(9%)
Transportation	3	(9%)
Environment	2	(6%)
Study Objective*, *n*(%)		
Substantive / empirical	32	(97%)
Educational / methodological	3	(9%)
Perspective*, *n*(%)		
Patient / consumer	18	(51%)
Provider / producer	13	(37%)
Citizen / societal	4	(11%)

*All that apply

**Table 2:** Design characteristics of BWS applications in Africa

	*N* = 35	
Methods of Instrument Development, * *n*(%)		
Literature review	26	(74%)
Formal qualitative methods	16	(46%)
Key informants	9	(26%)
Pretesting	7	(20%)
Piloting	6	(17%)
Prior preference research	6	(17%)
Mode of Survey Administration		
Administered	23	(66%)
Not specified	5	(14%)
Self-administered (paper/device)	4	(11%)
Online	3	(9%)
Time-horizon		
Present	31	(89%)
Future	4	(11%)
Past	0	(0%)
Measurement Scale		
Importance/priorities	24	(69%)
Preference	6	(17%)
Other	3	(9%)
Emotion (concern)	2	(6%)
BWS anchor description		
Most/Least	30	(86%)
Best/Worst	4	(11%)
Other	1	(3%)
Experimental Design		
BIBD	24	(69%)
Sawtooth	5	(14%)
Orthogonal	3	(9%)
Not specified	2	(6%)
Other	1	(3%)
Objects total mean, sd	15.9	(9.1)
Objects per task^#^ mean, sd	5.2	(3.5)
Choice tasks total^^^ mean, sd	22.4	(38.4)
Choice tasks per respondent^&^ mean, sd	13.7	(8.2)

*All that apply, ^#^*n* = 34, ^*n* = 31, ^&^*n* = 33, **BIBD = balanced incomplete block design

**Table 3:** Administration and analysis of BWS applications in Africa

	*N* = 35	
Sample Size, median (IQR)	282	(150–451)
Sample Size Justification, *n*(%)		
Not specified	29	(83%)
Formal sample size calculation	6	(17%)
Analytic Program*, *n*(%)		
Not specified	21	(60%)
Stata	5	(14%)
Excel	3	(9%)
SAS	3	(9%)
SPSS	3	(9%)
Sawtooth	2	(6%)
R	1	(3%)
Analytic Approach*, *n*(%)		
Counts	17	(49%)
B-W Score	16	(46%)
Probability/ratio rescaling	15	(43%)
Regression coefficients	15	(43%)
Square roots	5	(14%)
Theoretical Assumption, *n*(%)		
Not specified	16	(46%)
MaxDiff	9	(26%)
Simultaneous	5	(14%)
Sequential BWS	4	(11%)
Other	1	(3%)
Directionality, *n*(%)		
Bidirectional	23	(66%)
Unidirectional	12	(34%)
Heterogeneity Analysis used, *n*(%)	22	(63%)
Heterogeneity Analysis method*, *n*(%)		
Stratification	14	(40%)
Segmentation/latent class	7	(20%)
Mixed logit	4	(11%)
Individual level score	1	(3%)

*All that apply

## Results

The review identified 35 published studies using best-worst scaling for prioritization focused on (in full or in part) participants from Africa. As seen in Table 1, studies originated from northern Africa (*N* = 1), eastern Africa (*N* = 7), southern Africa (*N* = 13), and western Africa (*N* = 14). This included 7 studies not identified by previous reviews. The majority of the papers (72%) from Africa were published between 2019 and 2023 [40–63] and referred to ‘Best Worst Scaling’ in their write-up (85%) [40, 41, 43–47, 49, 51, 52, 54, 56–74]. Of the 14 countries where research was conducted, South Africa produced the most studies (34%) [41, 42, 46, 51, 52, 55, 56, 60, 61, 66, 67, 70, 74]. Most papers presented results from empirical research (97%) [40–42, 44–48, 50–68, 70–74]. Agriculture (43%) [41, 43–45, 47, 49–51, 54, 59, 60, 63–65, 69–71] and health (34%) [40, 46, 48, 52, 55–57, 62, 67, 72–74] were the most common research topics. Most studies (51%) measured preferences from the perspective of the patient / consumer [41, 42, 46, 49, 51, 54, 55, 57, 60, 63–70, 72], with nearly a third measuring provider / producer preferences (37%) [40, 43–45, 47, 48, 50, 56, 58, 59, 62, 71, 74].

### Study design

All identified studies articulated their approach to developing their BWS instrument. The vast majority, (74%), utilized a literature review [41, 42, 44–50, 52–57, 60, 61, 64–69, 71, 72, 74] while less than a quarter conducted piloting (17%) [42, 46, 50, 53, 56, 73] or pretesting (20%) [47, 50, 52, 54, 56, 66, 71] of the instrument prior to administration. Half of the studies reported utilizing formal qualitative methods during instrument development [40, 42, 43, 46, 48, 50, 53, 56, 57, 60, 62, 67–69, 73, 74]. In-person, surveyor-administered was the most common mode of survey administration (66%) [40, 43–48, 50, 52–55, 58, 59, 61, 62, 64–69, 72] with online (9%) [51, 56, 74] and self-administered (11%) [41, 57, 70, 73] less frequently utilized. The time horizon used to contextualize the survey was present tense most frequently (89%) [40–42, 44–53, 56–72, 74] with only four studies asking about the future (11%) [43, 54, 55, 73] and no studies asking about the past. BWS most frequently measured importance (69%) [41, 45, 46, 49, 51–53, 55–57, 59–61, 63, 64, 67–74], followed by preferences motivating responses (17%) [40, 43, 44, 48, 54, 65]. The most common phrasing to anchor the experiment was asking participants to choose the “most” and “least” [important/preferred/concerning] (86%) [40–42, 44–49, 51, 52, 54–57, 59–66, 68–74], followed by asking participants to choose the “best” and “worst” (11%) [43, 53, 58, 67]. The most common experimental design used was a Balanced Incomplete Block Design (BIBD) (69%) [40–46, 48–51, 53, 54, 56, 58–60, 64, 65, 67, 70–73] with 14% using a design from Sawtooth software [47, 57, 66, 68, 69]. The mean total objects per experiment was 15.9 (standard deviation (sd): 9.1, min 6: max: 48). Experiments had a mean of 5.2 objects per task (sd: 3.5, min: 3 max: 24), 22.4 (sd: 38.4, min: 1 max: 210) choice tasks per experiment and a mean of 13.7 (sd: 8.2, min: 1 max: 51) choice tasks per respondent during their participation in the experiment (Table 2).

### BWS administration and analysis

Median sample size was 282 participants (IQR: 150–451, min: 28, max: 1002) but only 17% of papers gave a formal sample size justification (Table 3) [40, 47, 48, 57, 62, 66]. Stata was the most reported data analysis program utilized (14%) [46, 50, 53, 67, 73], followed by Excel [42, 61, 66], SAS [43, 48, 62], or SPSS (9% each) [47, 66, 69]. The remaining studies (60%) did not specify which statistical analysis program they used. Probability / ratio rescaling (43%) [40, 42, 43, 45, 48, 49, 51, 54, 59, 60, 64, 65, 67, 68, 71], counts (49%) [40, 41, 45, 46, 50, 52–54, 56, 58, 61, 63, 66, 67, 70, 71, 73], regression coefficients (43%) [43, 44, 48, 49, 52, 53, 55, 63–65, 68, 69, 71–73], and best-worst scores (46%) [41, 42, 45–47, 52, 54, 58–61, 64, 66, 70, 71, 73] were common analysis approaches. Approximately half of the studies (40%) effects coded their data [40, 42, 49, 50, 52, 55, 64, 65, 69, 71, 72] while fewer (17%) used an omitted variable [43, 44, 48, 53, 54, 62]. Heterogeneity analyses were conducted by most studies (63%) [42–46, 49–52, 54, 55, 57–59, 64, 66–69, 72], with stratification being the most common heterogeneity analysis approach (40%) [42, 44, 49, 51, 52, 55, 57, 63–68, 72] followed next by latent class analysis (20%) [45, 46, 50, 54, 58, 59, 69].

### Study quality and policy relevance

Policy relevance of studies was high with 66% scoring 7 or above on the 10-point scale [40–42, 44, 46, 48–55, 57, 59, 64, 65, 67, 68, 70–73]. Most studies scored in the upper half of the PREFS scale [3–5, 40, 42, 44–54, 57–60, 62–72].

### Strengths and weaknesses of BWS

Strengths and weaknesses of using BWS were identified in most of the published studies from Africa. Most of these studies focused solely on strengths of using BWS, though some identified both strengths and weaknesses and a few focused solely on weaknesses. These strengths and weaknesses were categorized into seven domains: context, purpose, method, burden, results, comparisons, and bias (Table 4).

**Table 4:** Strengths and weaknesses of BWS

Domains	Strengths	Weaknesses
Context	Populations with lower education levels [52, 72]; Low-income [72, 73]; Living in urban areas [66]	Difficult to apply in field work situations [57]; Lower education levels [52]
Purpose	Engage communities [42]; Inform decision-making [55]	
Method	Good alternative to other ranking methods [41, 42, 59, 61, 71]; Simplicity [49, 58, 66, 68]; Theory-based with established literature [69, 71, 72]; Flexibility in analytical methods [50, 53, 71]	Hypothetical scenarios [64]
Burden	Reduces respondent burden [41, 45, 49, 50, 52, 55, 59, 61, 66, 68, 72]; Stakeholder burden [42, 45, 52, 65]; Investigator burden [52]	Possible respondent burden [52, 59, 66]
Results	More information captured (generally) [49, 59, 64, 66, 68, 72]; Quality and precision [41, 45, 49, 58, 71]; Ratio scale [45, 51, 64, 70]; Individual level scores [50, 64]; Relative importance [41, 60]; Understanding respondents choice processes [55, 66]	
Comparisons	Discrimination between objects [45, 49, 59, 65, 68, 73]; Trade-offs [45, 50, 59, 68]; Populations [51, 70]	Between population comparisons [68, 69]
Bias	General bias [41, 51, 53, 59, 66, 70]; Scale bias [56, 71]; Response bias [56]	Desirability bias [72]

Strengths were noted across all seven domains. In the context domain, BWS was noted for eliciting priorities and preferences from populations with lower education levels [52, 72] and lower income [72, 73]. The purpose domain highlighted strengths such as engaging communities [42] and informing decision-making [55]. The method domain emphasized that BWS overcomes limitations of other ranking methods [41, 42, 59, 61, 71], including variations in interpretation related to Likert-type scales. The burden domain commonly cited that BWS reduces respondent burden [41, 45, 49, 50, 52, 55, 59, 61, 66, 68, 72]. The results domain stressed that BWS captures more information [49, 59, 64, 66, 68, 72] and produces higher quality and more precise results than other methods [41, 45, 49, 58, 71]. The comparisons domain focused on the ability to discriminate between objects [45, 49, 59, 65, 68, 73]. The bias domain noted a reduction of general bias [41, 51, 53, 59, 66, 70].

Weaknesses or limitations of using BWS were provided for only five of the seven domains. In the context domain, it was suggested that BWS might be challenging to use in clinical practice as a decision-making tool [57] and, contrary to studies noting it as a strength, some identified it as, challenging for populations with lower education levels [52]. The method domain pointed out that BWS involves hypothetical scenarios that may not be realistic [64]. The burden domain cited possible respondent burden associated with completing a series of BWS tasks [52, 59, 66]. The comparisons domain highlighted potential difficulties in making comparisons between populations within the sample [68, 69]. The bias domain noted the possibility of desirability bias, where respondents report socially acceptable factors rather than genuine preferences [72]. Most of these weaknesses were posed as possibilities, rather than observed limitations.

### Case studies

*Policy relevance*: Ozawa et al. [72] used BWS scaling to inform message development and effective delivery strategies with the goal of improving childhood vaccination awareness and demand in Nahuche, Zamfara State northern Nigeria, a region with low vaccination uptake. *Instrument development*: The survey items were developed through a review of published literature from Nigeria and other low-and-middle income countries on factors that affect childhood vaccine demand and uptake. Identified factors were categorized into four groups and each written out as a negative or positive influence based on the literature (e.g., vaccines may harm a child (negative), trust the views of leaders about vaccines (positive)), balancing equal numbers of positive and negative statements. A local study advisory board reviewed the items. *Population*: The survey was administered in-person to parents with children under 5 years old during a household survey from a representative sample of households. *Administration*: The survey was translated into Hausa and presented as a pictorial questionnaire with both photographs and words used to represent each factor. *Perspective and time scale*: Participants were asked to select the most and least important factors to them (consumer/patient) when deciding whether to vaccinate a 1-year-old child (present). *Design*: The study utilized a BIBD where every participant was presented with 16 choice sets of 6 factors. The survey took approximately 1 h to complete. *Analysis*: They assumed sequential BWS and used conditional logistic regression with effects coding to determine factor rankings. *Heterogeneity*: They examined heterogeneity in the results by looking at different strata: male/female parent and by previous diphtheria, tetanus, and pertussis (DTaP) vaccination status (yes/no). *Participation*: 198 parents participated. *Results*: The most important motivating factor for vaccinating children was the perception that vaccination makes one a good parent. Trust and norms were found to be more important than benefits and risks in vaccination decisions. They identified differences in rankings between fathers and mothers and in families with and without prior DTaP vaccination.

*Policy relevance*: Yemeke et al. [52] compared the Uganda national budget resource allocations across 16 sectors to citizen preferences for such allocations. A particular emphasis was placed on understanding the health care sector as a funding priority. *Instrument development*: The sixteen survey factors represented each of the sixteen sector allocations within the Uganda national government’s budget. Results from a pre-test of the survey to assess respondent understanding were used to improve the instrument. *Population*: The survey was administered in-person to the head of household or spouse, of at least 18 years of age, in both rural and urban areas in the Mukono district in central Uganda. *Administration*: The survey was translated into Luganda and displayed accompanying pictorial representations (such as photographs or graphics) of the sectors. Descriptions of the sectors and their functions were also read aloud. *Perspective and time scale*: Respondents were asked to select the most and least important sectors for resource allocation for their community (present) in each choice task, offering societal perspective prioritization. *Design*: A main effects orthogonal design was used to generate 16 choice tasks, each with 4 sectors (factors). *Analysis*: Count analysis: Relative mean best-worse scores were calculated for each sector. Scores were transformed to a positive scale, anchored at zero, to calculate percentage preference relative to estimated cumulative sums. The preferred percentages were compared to the actual percentages allocated in the national budget. Assuming sequential BWS, the authors used McFadden’s conditional logistic regression with effects coding to regress a single dichotomous choice variable on all sectors to assess ranking of preferred sectors. *Heterogeneity*: They examined heterogeneity in the results across two strata (urban/rural). *Participation*: There were 432 respondents across two settings (217 urban respondents, 215 rural respondents). *Results*: The health sector was the highest ranked sector by a significant margin amongst both rural and urban respondents. This result was consistent in both the relative best-minus-worst score method and the regression analysis. This highlighted a clear disparity between citizen preferences and national budget al.location, where the health sector was ranked sixth.

*Policy relevance*: Nyarko et al. [48] used BWS to quantify the antimicrobial dispensing practices of medicine sales outlet staff. Understanding these practices can help to improve patient safety and care quality, as well as to serve as a guide for decision-making in the pharmaceutical sector. *Instrument development*: The initial list of survey items was identified through informant interviews with medicine sales experts and an extensive literature review. The list of items was condensed into eight objects through focus group discussions with medicine sales outlet staff. *Population*: The survey was conducted in-person through interviewer-questionnaire administration with medicine sales outlet staff over a two-month period. Staff were eligible for the study if they had dispensed microbials within the past year. *Administration*: Demographic information was collected at the start of the questionnaire, followed by questions regarding the staff’s prescribing and dispensing practices of antimicrobial medications. *Perspective and time scale*: Participants were asked to indicate which object regarding antimicrobial dispensing practices concerned them the most and least. *Design*: A BIBD was used, generating 8 tasks, each with 7 objects. *Analysis*: Assuming the respondent chose the items they most liked and disliked, a maximum difference model with effects coding was used to determine parameter estimates. The relative importance of each object was determined based on the parameter estimates, allowing the objects to be ranked by level of importance. *Heterogeneity*: Heterogeneity was examined by comparing antimicrobial dispensing practices with their associated objects. *Participation*: 200 staff participated. *Results*: The antimicrobial dispensing practice that concerned respondents most was the need to follow the drug act and avoid dispensing antimicrobials without a prescription. Dispensing antibiotics to poor patients who may not be able to afford medical bills was not a concern for respondents. Overall, the study suggests that staff are careful when dispensing antimicrobials.

## Discussion

Our study identified 35 studies from across Africa, with the majority published in the past four years. BWS has most commonly been used in agriculture and health, although its broad applicability is demonstrated through its use in fields including business, environment, and transportation. Published studies from eastern, western, southern, and northern Africa include a broad range of sample sizes, design choices, and analytical approaches. Consistent with other BWS reviews [25], the majority of studies are both of high quality and of high policy relevance. It is interesting to highlight that among articles classified as ‘multi-country,’ two articles included participants from at least one African country in their sample. However, we considered them ‘near misses’ and excluded them as neither disaggregated data by country to ensure review data came from Africa. Both had few participants from African countries relative to the overall sample.

The application of BWS for prioritization in the Africa context is an emerging practice as demonstrated by our findings that its use has increased dramatically over time. The quality of studies, as measured by PREFS, has remained consistently high over time [24]. As the method continues to be applied, guidelines exist that could further ensure researchers conduct high-quality studies and publish high-quality papers about them [38, 39, 75]. This includes the increased use of instrument development methods, especially formal qualitative work, pretesting with cognitive interviewing, and piloting [76]. Importantly, this also requires attentiveness to ensuring accuracy of conceptual translation which is often missed if focusing exclusively on linguistic translation for studies working across multiple language groups [77].

The use of BWS also draws attention to the notion of prioritization itself. Our findings highlight the relevance of the method to policy making; over three-quarters of the included studies received a policy relevance score of seven or more. Clearly expressed priorities may allow policy makers to shift away from informal decision-making heuristics to more formal, principled decision making practices. Certainly, the more recently observed integration of BWS into deliberative processes [78] such as the modified policy Delphi [79] and deliberative democracy [80] exemplifies other ways that priority and preference elicitation can help inform group deliberation to achieve consensus [81, 82]. That said, there remain questions about aggregating individual priorities in group decision making [36] and is a topic that others have grappled with, including in health state valuation [83]. Finally, it is important to note that BWS is only one method to assess priorities, where other methods include deliberation [84], simple rating or ranking approaches alone or as a part of a Delphi approach [85], pick n of m tasks [86], or stated preference methods such as willingness to pay [87], DCEs, or conjoint analyses [88].

With only 34% of included studies in health, this shows an opportunity for greater application in the health field. The range of health-specific topics to which BWS was applied in this study demonstrates versatility across health areas. Various types of preference facilitation have proven successful in health-specific areas [25, 89]. Additionally, as health systems are conceptualized more broadly to include social determinants of health such as transportation options, food systems, and the environment the other topical applications demonstrate direct relevance to health systems and decision-making. Similarly, its use in business may be applicable to business-based approaches to health such as social marketing strategies for behavior change. While patient-centered care may involve individual-level preference accommodation of individual patients (e.g., choosing a community-adherence group over fast-track appointments among HIV differentiated service delivery options), systematic understanding of trends in patient preferences at a broader level can inform efficient health system decision-making (e.g., the creation of differentiated service delivery options for HIV). The two studies including data from an African country that we nearly included but did not due to lack of geographic specificity both show successful implementation of multi-country BWS.

Our study is subject to publication bias, as the systematic review searched published literature. The review targeted object case BWS (also known as case 1, MaxDiff, and object scaling). Further research into other types of BWS would contribute to a more comprehensive understanding of BWS in Africa. While we introduce BWS, we do not offer guidance on methods or analysis. Multiple other peer reviewed papers, including some included in this review [42], and texts offer clear instruction on BWS implementation to aid researchers wishing to apply this method [36]. Further, we do not include assessments of BWS participation from the participant perspective, as literature on this topic is very limited [90]. The field would benefit from frameworks for participant assessment of BWS participation to better incorporate this into BWS findings, as have been developed for instrument development [76].

## Conclusion

We need effective tools to measure preferences and priorities, including tools that suit those whose input is more traditionally sought in health decision making (e.g., providers) and those whose voice is critical, but often unheard (e.g., patients, consumers, and community members). BWS is one of those tools. BWS offers a versatile alternative to DCEs and other better-known methods of measuring preferences. Researchers can successfully employ BWS across a range of sample sizes, and using various analysis approaches and programs. Growing use of the method across the African continent demonstrates its feasibility and utility, recommending it for consideration among researchers, program implementers, policy makers, and funders when conducting preference research to influence policy and improve health systems. Further research can help to recommend specific applications, including further work to understand context-specific implications of the strengths and limitations of the methods alongside cognitive burden and population-specific recommendations.

## Data Availability

All study data are available upon request from the authors for the review registered on PROSPERO (CRD42020209745).

## Abbreviations

BIBD: Balanced incomplete block design
BWS: Best-worst scaling
DCE: Discrete choice experiments
DTaP: Diphtheria, tetanus, and pertussis
PREFS: Purpose, responses, explanations, findings
PRISMA: Preferred Reporting Items for Systematic Reviews and Meta-Analyses
